# Imaging of developing human brains with ex vivo PSOCT and dMRI

**DOI:** 10.1162/imag_a_00510

**Published:** 2025-03-24

**Authors:** Hui Wang, Nathan Blanke, Dayang Gong, Alpen Ortug, Jose Luis Alatorre Warren, Christopher Clickner, William Ammon, Jackson Nolan, Zoe Cotronis, Andre van der Kouwe, Emi Takahashi

**Affiliations:** Athinoula A. Martinos Center for Biomedical Imaging, Department of Radiology, Massachusetts General Hospital, Charlestown, MA, United States; Harvard Medical School, Boston, MA, United States; Center for Lifespan Changes in Brain and Cognition, University of Oslo, Oslo, Norway; College of Science, Northeastern University, Boston, MA, United States; Lurie Center for Autism, Massachusetts General Hospital, Lexington, MA, United States

**Keywords:** diffusion MRI, myelination, neurodevelopment, neuroimaging, Polarization-Sensitive Optical Coherence Tomography (PSOCT), white matter anatomy

## Abstract

The human brain undergoes substantial developmental changes in the first 5 years of life. Particularly in the white matter, myelination of axons occurs near birth and continues at a rapid pace during the first 2 to 3 years. Diffusion MRI (dMRI) has revolutionized our understanding of developmental trajectories in white matter. However, the mm-resolution of*in vivo*techniques bears significant limitation in revealing the microstructure of the developing brain. Polarization sensitive optical coherence tomography (PSOCT) is a three-dimensional (3D) optical imaging technique that uses polarized light interferometry to target myelinated fiber tracts with micrometer resolution. Previous studies have shown that PSOCT contributes significantly to the elucidation of myelin content and quantification of fiber orientation in adult human brains. However, the use of PSOCT in developing human brains has not been reported. In this study, we established the feasibility of using the PSOCT technique to reveal brain development during the first 5 years of life, compared with ex vivo dMRI. The results showed that the optical properties of PSOCT quantitatively reveal the myelination process in young children. The imaging contrast of the optic axis orientation is a sensitive measure of fiber orientations in largely unmyelinated brains as young as 3 months old. The micrometer resolution of PSOCT provides substantially enriched information about complex fiber networks and complements submillimeter dMRI. This new optical tool offers great potential to reveal the white matter structures in normal neurodevelopment and developmental disorders in unprecedented detail.

## 1 Introduction

Myelin, which surrounds the axonal fibers, plays a crucial role in brain function by facilitating rapid and coordinated neuronal communication throughout the brain. Postnatal myelination occurs most rapidly within the first 2 years of life (Dobbing & Sands, 1973;Matsuzawa et al., 2001). During the first year, the volume of white matter in the human brain increases by 6–16%, primarily due to myelination (Grotheer et al., 2022). This time window is critical for maintaining a healthy developmental trajectory of neuroanatomy and physiology (Turesky et al., 2020). Disorders of myelination such as multiple sclerosis, Guillain-Barré syndrome, and many others have been associated with a variety of developmental and cognitive impairments (Grotheer et al., 2022). MRI has revolutionized the way myelination is assessed in young children. Prior to the use of MRI, the only possible way to determine the developmental status of myelination was by clinical course, neurological examination, and histological examination (Welker & Patton, 2012). Diffusion MRI (dMRI) sequences use diffusion-sensitized gradient pulses to probe the anisotropic diffusion of water molecules in brain tissue, which allows imaging of pathways with coherent water diffusivity, including developing axons. Fractional anisotropy (FA), which indicates the degree of anisotropy of water diffusivity, serves as a good indicator for white matter organization, because coherent axonal organization restricts the movement of water molecules perpendicular to the axon bundle (Welker & Patton, 2012). A link between FA increase and myelin maturation in infants has been suggested (Hermoye et al., 2006;McGraw et al., 2002;Mukherjee et al., 2002). In addition, apparent diffusion coefficient (ADC) has been found to decrease through early childhood, due to more restricted diffusion of water molecules across the myelin sheath (Sotardi et al., 2020). However, rapid changes in brain anatomy and physiology pose many distinct limitations to the acquisition, data processing, analysis, and interpretation of these developmental trajectories (Turesky et al., 2020). It is known that dMRI tractography has difficulty identifying complex fiber configurations from multiple directions in complex structures (Schilling et al., 2019). This problem becomes more prominent in the developing small brain.

Optical imaging methods provide superior resolution compared to MRI techniques. Among them, polarization sensitive optical coherence tomography (PSOCT) provides label-free and depth-resolved imaging contrasts that originate from light scattering and tissue birefringence (De Boer et al., 1997). When applied to brain imaging, the intensity contrast from PSOCT reveals the gross anatomical structure and provides quantification of the scattering coefficient (H. Wang et al., 2017;Yang et al., 2020). Birefringence originates from the structural anisotropy of myelinated fibers and results in an optic axis that is parallel to the fiber orientation (H. Wang et al., 2011). Fiber tracts as small as tens of micrometers in diameter can be resolved with the retardance map, and the fiber orientation can be measured by the optic axis orientation (Liu et al., 2022;H. Wang, Zhu, & Akkin, 2014). The optical properties of both scattering coefficient and birefringence are high in myelinated fibers and low in unmyelinated fibers. In addition, retardance provides a measure of the degree of alignment in myelinated fibers across the imaging depth, in which crossing fibers reduce the value of retardance. The development of automatic serial sectioning PSOCT (as-PSOCT) based on blockface imaging has proven to be a valuable tool for mapping the complex and intricate white-matter organization in the human brain (H. Wang et al., 2018) (also see a recent reviewYendiki et al., 2022). By integrating a tissue slicer into the imaging system, as-PSOCT has proven to be an effective way to map fiber orientations across cubic centimeter specimens of human brain. One advantage of as-PSOCT is that it does not suffer from the nonlinear distortions plaguing slice-based histological techniques that demand complex registration frameworks to correct (Ali et al., 2018;Majka & Wójcik, 2016). As a result, the as-PSOCT technique allows for the reconstruction of large volumes of brain samples with microscopic-level resolution. To date, PSOCT has been used to study adult human brains. The usefulness of PSOCT in infancy and early childhood, when myelination is in process, has not been reported.

In this work, we leveraged the capability of as-PSOCT to investigate the neurodevelopment of the human brain in early childhood, compared with dMRI results. We found that as-PSOCT was sensitive in delineating the myelination process in the infant brain as early as 3 months old. Optical properties revealed distinctive developmental patterns in different brain regions across the first 5 years. Correlations between as-PSOCT and dMRI images showed general agreement in fiber tracts, while the optical imaging resolved finer details on tissue properties and fiber orientations. The high resolution of the as-PSOCT technique offers great potential for studying typical and atypical neurodevelopment in the human brain.

## 2 Method

### 2.1 Samples

Five human brain samples (3 males, 2 females) were obtained in coronal slabs from the University of Maryland Brain and Tissue Bank (UMBTB) through the NIH NeuroBioBank (NBB) network. All specimens were collected by the UMBTB and NBB between 2009 and 2016 and subsequently stored in formalin. The slabs were approximately 2 cm thick and fixed in 10% formalin when received. The brain samples had different developmental timelines: 3, 6, 15, 50, and 54 months old. One subject was diagnosed with sudden infant death syndrome (3 months old), one with autism spectrum disorder (54 months old), and the other three were controls without neurological disease. The postmortem intervals were less than 40 h.Supplementary Table S1describes the detailed individual information about the specimens.

### 2.2 Ex vivo diffusion MRI and data processing

Slabs of the postmortem brains were prepared for MRI scanning by soaking in bags containing fomblin oil (same used as, e.g.,Ortug et al., 2023;Takahashi et al., 2012). These bags were put in a container for each subject, arranged side by side with plastic plates dividing them. The scans were performed at the Athinoula A. Martinos Center for Biomedical Imaging for all 5 brain samples. The primary obstacle to conducting high-resolution ex vivo human dMRI studies is the significantly reduced T2 and diffusivity of fixed tissue (Roebroeck et al., 2019). To address these challenges, we adjusted the acquisition parameters by referring to the previous literature on fixed human brain dMRI, and further optimized them based on our previous study (Ortug et al., 2023). Diffusion-weighted MRI data were acquired using a diffusion-weighted SSFP sequence (McNab et al., 2009;McNab & Miller, 2010) on a 3 Tesla Siemens Trio scanner. The following parameters were used: TR = 28.82 ms, TE = 24.42 ms, flip angle = 35°, resolution = 0.8 mm isotropic, in-plane FOV = 144 mm × 144 mm, number of slices = 88, and bandwidth = 149 Hz/px. Diffusion weighting was performed along 60 directions with 10 T2-weighted b = 0 measurements. In diffusion-weighted SSFP acquisitions, the b-value varies with tissue T1 and T2 relaxation times. In our experiment, with diffusion gradient duration of 20 ms, the b-value was approximately 4,000 s/mm^2^. Total scan time was 6 h 11 min 43 s.

Diffusion Toolkit (R. Wang et al., 2007) and TrackVis (Version 0.6.1; trackvis.org) were used to reconstruct and visualize tractography. The FACT algorithm and 60˚-angle thresholds were used in the diffusion tensor imaging (DTI) model to reconstruct tractography pathways. No FA threshold was applied (Shiohama et al., 2022). As multiple brain slabs were scanned together in a box, after tractography was performed in the entire FOV, individual slabs were segmented using Amira software (Stalling et al., 2005) (3D Version 2021.2, Thermo Fisher Scientific, Waltham, MA, USA), and were analyzed separately.

### 2.3 PSOCT imaging and data processing

#### 2.3.1 PSOCT system

One coronal slab per brain was embedded in agarose using a customized grater (Chang et al., 2023) and refractive index matched in either 30% TDE or 50% glycerol for PSOCT imaging. The anatomical locations of the coronal slabs scanned using both modalities were labeled inFigure 1. A home-made automatic serial sectioning PSOCT (as-PSOCT) system was used for data collection. The system integrates a commercial spectral domain PSOCT system (TEL220PS, Thorlabs), motorized xyz translational stages, and a vibratome tissue slicer. Custom-built software, written in C++, provides coordinated data acquisition, xyz-stage translation, and vibratome sectioning for automatic imaging of brain blocks. The maximum sensitivity of the PSOCT was 109 dB. The imaging depth was 2.6 mm with an axial resolution of 4.2 μm in tissue. The samples were imaged with a scan lens objective (OCT-LSM3, Thorlabs), yielding a lateral resolution of 10 μm. One volumetric acquisition was composed of 350 A-lines and 350 B-lines covering a field of view (FOV) of 3.5 x 3.5 mm at an A-line rate of 50 kHz. We imaged the block face surface of the entire coronal section via tile scans with a 20% overlap. Two of the five samples (50 and 54 months old) went through serial sectioning to acquire large volumetric image sets. A 100–150 µm thick slice was removed from the tissue surface by the vibratome to expose the deeper region until the whole block of tissue was imaged.

**Fig. 1.**
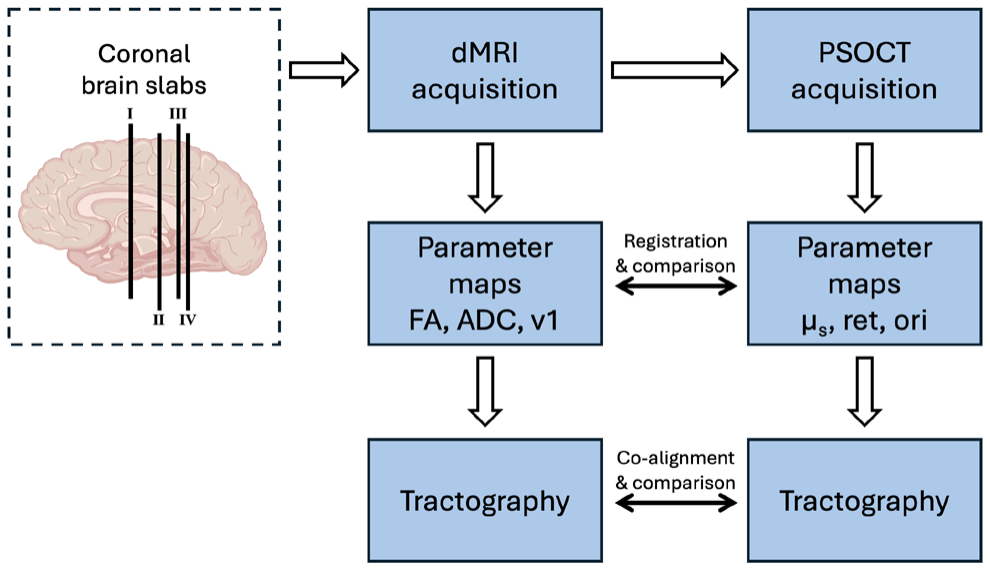
Pipeline of image acquisition and processing. The anatomical location of each coronal slab is overlaid on the cartoon diagram of the brain.

#### 2.3.2 Image reconstruction

Inverse Fourier transform of interference-related spectral oscillations yielded complex depth profiles in the form of,$A_{1,2}\left(z\right)\exp\left[i\phi_{1,2}\left(z\right)\right]$whereAandϕdenote the amplitude and phase as a function of depthz, and the subscripts represent the polarization channels. The image contrasts of intensityA(z),R(z), retardance,δ(z), and optic axis orientation,θ(z)along depth, were obtained by$R\left(z\right)\propto A_{1}{\left(z\right)}^{2}\;+A_{2}{\left(z\right)}^{2}$,$\delta \left(z\right)=\arctan\left[A_{1}\left(z\right)/A_{2}\left(z\right)\right]$and$\theta \left(z\right)=\left[\phi_{1}\left(z\right)-\phi_{2}\left(z\right)\right]/2$, respectively. We also quantified the voxel-wise attenuation coefficient following the method of Vermeer et al. In the near-infrared spectral range, light attenuation within the tissue is dominated by scattering, whereas absorption is negligible. Therefore, we used the scattering coefficient (${µ}_{s}\left(z\right)$), calculated as,${µ}_{s}\left(z\right)=I\left(z\right)/\left[2\Delta \sum_{j=z+1}^{d}I\left(j\right)\right]$to represent the attenuation coefficient, wherezis the pixel number in depth,Iis the reflectivity signal,Δis the pixel size in depth, anddis the total imaging depth in mm (Vermeer et al., 2013). En-face scattering coefficient and retardance images were calculated by averaging the respective values along the slice thickness. The orientation at each pixel in the en-face axis orientation images corresponded to the peak of a histogram constructed by binning the measured orientation values into 5° intervals. We stitched the tiles to reconstruct the entire section in the Fiji software which computed the overlap between tiles and used linear blending for image fusion (Preibisch et al., 2009). In the two samples with serial sectioning, the images of individual slices were stacked together to render the volumetric reconstruction of the coronal slab.

#### 2.3.3 Tractography

Tractography was applied to the in-plane optic axis orientation images using the conventional method for DTI modeling in Diffusion Toolkit. The optic axis orientation was used to track the fibers, while the orientation for the z-direction was set to 0. The fiber tracking algorithm is based on the spherical harmonic basis method (Hess et al., 2006). Tracts were created with a maximum angular threshold of 45° for tracking and masked by the retardance image to include the white matter only.

### 2.4 PSOCT-dMRI registration

We co-registered PSOCT and dMRI data for a further correlation and comparison study. For samples that only had one PSOCT imaging section, we found the closest dMRI slice to the PSOCT images and manually rotated the parameter images and the tractography to match the anatomical directions. For samples that had PSOCT with serial sectioning, we used robust registration, an automatic registration method that is insensitive to outlier areas of the images (Reuter et al., 2010), to register volumetric PSOCT and dMRI. We aligned the dMRI and PSOCT data from each block by registering the ADC map to the scattering coefficient map, as they possess the best gray/white matter contrast in the younger ages. The absence of non-linear distortions is a key feature of the PSOCT acquisition that facilitates this step, in comparison to alternative histological techniques. Thus, an affine registration was sufficient for the cross-modal alignment (H. Wang, Zhu, Reuter, et al., 2014). To evaluate the volumetric registration, we created a tissue mask and white matter mask for both PSOCT and dMRI volumes and computed the Dice coefficient (Dice, 1945) between the two modalities.

For the dMRI orientation registration, we applied the rotational component of the affine transformation to the dMRI orientation vectors and extracted the corresponding in-plane orientation. This allowed voxel-wise correlation of the diffusion-based orientation with direct measurements of axonal orientations from PSOCT. We also compared the PSOCT orientation with dMRI tractography.

### 2.5 Correlation between PSOCT and dMRI

To quantitatively evaluate the correlation of the metrics between PSOCT and dMRI, we conducted two analyses. For the first analysis, we manually segmented the white matter in both PSOCT and the corresponding dMRI slice and compared mean metrics across the entire white matter. For the second analysis, we manually selected 0.9 × 0.9 mm ROIs that were evenly distributed in the white matter of corresponding PSOCT and dMRI images. For PSOCT, we calculated the mean scattering coefficient, retardance, and circular mean (Berens, 2009) optic-axis orientation of each ROI. For dMRI, we calculated the mean ADC, FA, and circular mean diffusion orientation of each ROI. It is noted that the dMRI orientation vector was mapped into the PSOCT imaging plane first before obtaining the circular mean diffusion orientation. We then correlated the optical properties and dMRI parameter metrics using a linear fitting tool, both within the sample and across samples of different ages, and we report the Pearson’s correlation coefficient (r) values. For quantitative comparison of orientation, we investigated the angular difference between the two modalities and displayed them in a polar plot. The full pipeline of data acquisition and image processing is summarized inFigure 1.

## 3 Results

### 3.1 PSOCT showing myelin development in the first 5 years

**Fig. 2.**
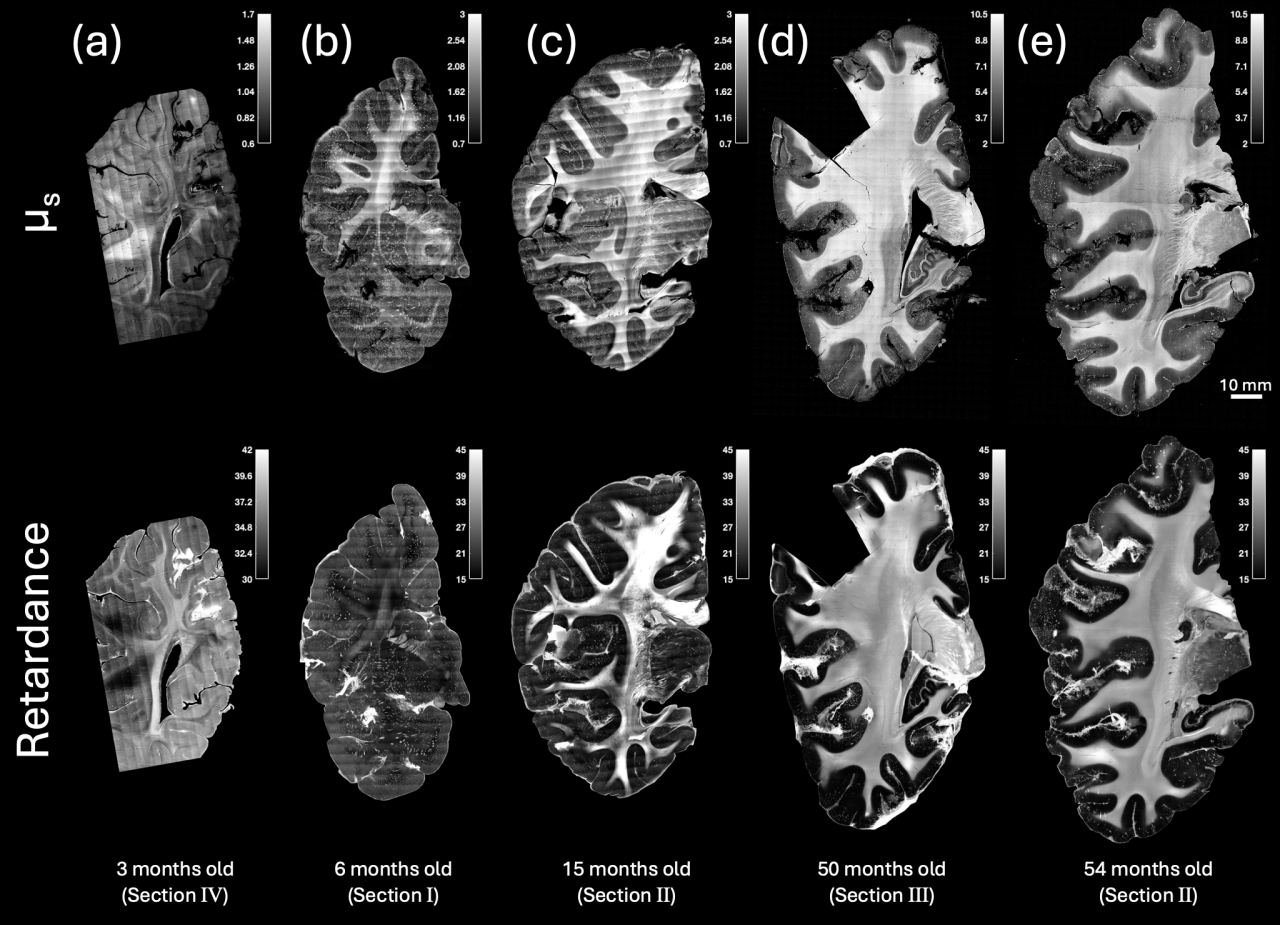
PSOCT optical property maps of scattering coefficient and retardance showing brain development in the first 5 years. (top) Scattering (μ_s_) maps and (bottom) retardance maps are shown for five samples from infancy to early childhood: (a) 3 months old, (b) 6 months old, (c) 15 months old, (d) 50 months old, and (e) 54 months old. The scale for each grayscale image is shown in the top-right inset of each panel. The anatomical location of each slab is denoted by Roman numerals and is shown inFigure 1.

**Fig. 3.**
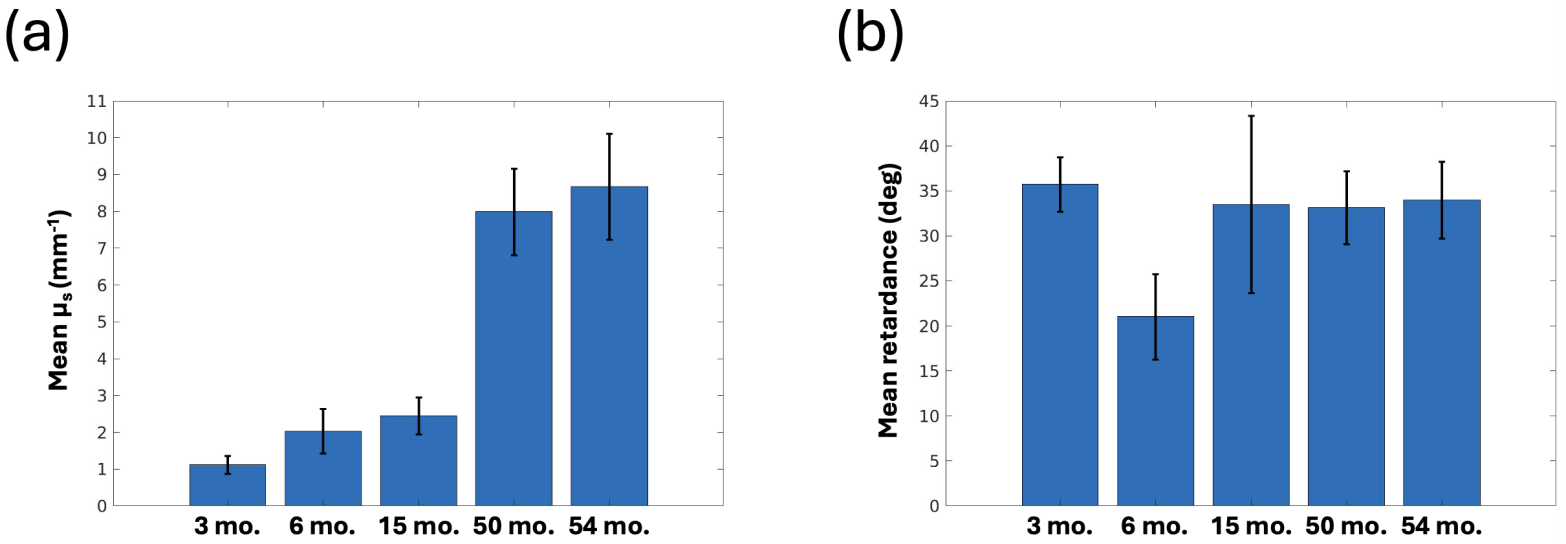
Change of PSOCT optical properties with white matter development. (a) Mean scattering coefficient (μ_s_) and (b) mean retardance analyzed across all pixels in white matter. The error bars show standard deviation.

The optical properties of PSOCT reveal detailed structural components in the brain. It is known that both scattering coefficient and birefringence are correlated with the myelin content. In this study, we show that the optical properties of PSOCT reveal the myelination process in young children. The scattering coefficient in white matter increases with age (Fig. 2, top row), indicating myelin maturation. At 3 months of age, the contrast between white and gray matter is not outstanding (a), whereas by 6 months of age, white matter starts to show a higher value than gray matter in some brain regions (b). It is interesting to observe the regional heterogeneity of myelination during infancy. In the anterior part of the brain (section I), the border of the parietal/frontal lobes (somatosensory/motor cortices) shows a higher scattering coefficient than the temporal lobe, indicating that myelination is advanced in the superior part of the anterior section at 6 months of age. This regional difference becomes invisible in the posterior brain by 15 months of age (c). The scattering coefficient in the white matter continues to rise substantially in the next 4 years (d and e). In contrast, the scattering coefficient stays low in the gray matter despite a small increase across multiple years. Quantitative analysis shows that the value increases almost 10 times in the white matter across the first 5 years of life (Fig. 3a). The retardance maps present more varying contrasts both within and across subjects, with white matter values higher or lower than gray matter (Fig. 2, bottom row). One possible contributing factor is the heterogeneity of optic axis orientation within the fiber bundles across the imaging depth. In regions where multiple fiber tracts meet and where fibers are oriented through the imaging plane, a reduced retardance is observed due to signal cancellation at the oppositely oriented myelin optic axes (Axer, Amunts, et al., 2011;Blanke et al., 2023). It is also noted that the retardance of the 6-month-old sample is lower in the white matter than the other samples, accompanied by numerous bright spots surrounding the fiber tracts. This is likely attributable to poor tissue quality, where the under-developed myelin is vulnerable at this young age. For comparison, corresponding parameter maps (Supplementary Fig. S1) and bar plots (Supplementary Fig. S2) are also provided for dMRI ADC and FA. We found that there is a general agreement between the optical property and dMRI scalar maps both visually and in the bar plots. For example, the mean FA value was low in the 6-month-old sample (Supplementary Fig. S2b) and the superior-inferior intensity gradient was also observed in the ADC and FA maps (Fig. S1b). It is noticeable that the ADC was low in the 3-month-old sample compared to 6- and 15-month-old. This trend is consistent with our other data not presented in this study.

### 3.2 Macroscopic and microscopic fiber pathways imaged with PSOCT and dMRI

**Fig. 4.**
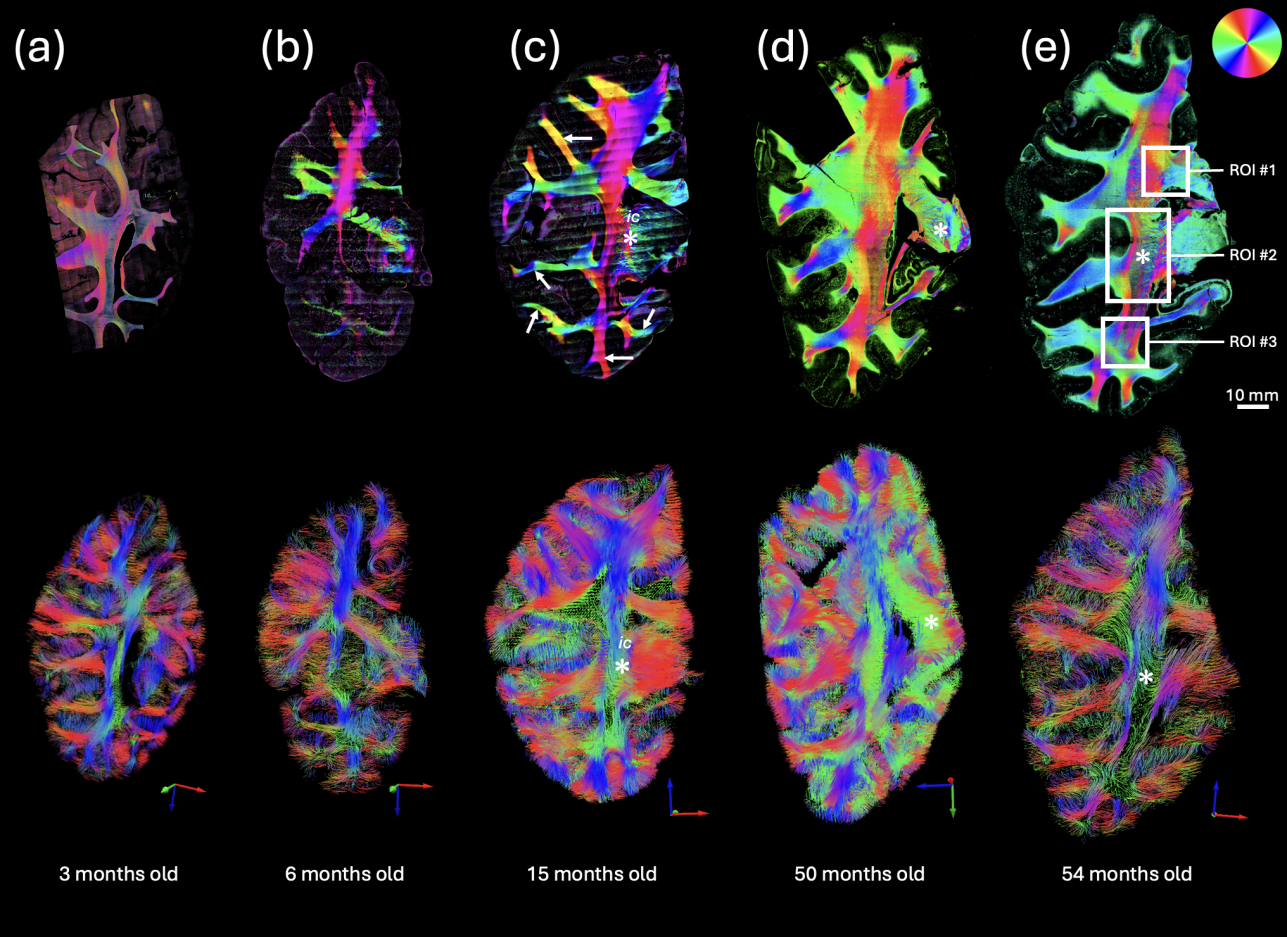
Fiber pathways visualized with PSOCT optic axis orientation maps and dMRI tractography. (top) PSOCT optic axis orientation maps and (bottom) dMRI tractography visualizations are shown for five samples from infancy to early childhood: (a) 3 months old, (b) 6 months old, (c) 15 months old, (d) 50 months old, and (e) 54 months old. The color scale used for showing the fiber orientation based on PSOCT optic axis is shown in the top-right corner of (e). The color axis used to display each dMRI tractography view is shown in the bottom-right of each inset. Three regions of interest are indicated in (e) that were selected for closer inspection inFigure 5.

The myelin sheath around axons presents an optic axis that is normal to the fiber orientation (Bear et al., 1997;de Campos Vidal et al., 1980), and that anisotropic material property provides the ability to map the in-plane fiber orientation with PSOCT optic axis orientation maps. The optic axis orientation of fiber pathways has been well-studied in adult human brain with PSOCT and other polarization microscopy techniques (Axer, Amunts, et al., 2011;Axer, Grässel, et al., 2011;Blanke et al., 2023). Here, we show that the optic axis enables fiber orientation measurements during the first 5 years of life (Fig. 4, top row). As indicated by the color wheel, the major fiber bundle orientations seen in multiple samples are consistent across different ages. Large fiber bundles of the internal capsule running along the superior-inferior axis show red-magenta colors, while tracts extending left and right into different cortical regions show colors of green, blue, and yellow. It is noted that the low myelinated white matter regions show greater noise in the orientation measurement. The optic axis orientation in the 3-month-old (Fig. 4a, top) only manifests in major fiber bundles that share orientation in the plane, and the overall noise level is higher than in the rest of the samples. At 6 months of age (Fig. 4b, top), the SNR in the temporal lobe is lower than in the parietal lobe, likely due to different myelin levels. Despite the differences in noise levels, our PSOCT technology proved sensitive in capturing fiber orientation in infants as early as 3 months of age.

In each sample, the dMRI tractography was aligned with the PSOCT imaging plane for comparison. Overall, the dMRI-based tractography was consistent with the PSOCT-based orientation. For example, the main axis of the internal capsule (ic) is superior-inferior (Fig. 4), and fibers toward the outer gray matter are oriented either left-right or superior-inferior (white arrows), depending on location. dMRI maintains a high quality of tract identification in the younger samples at 3 months old (Fig. 4a, bottom row) and 6 months old (Fig. 4b, bottom row). In contrast, PSOCT detected more detailed and distinct fiber orientation in samples at 15 months old and older (Fig. 4c–e, asterisks). For further comparison, dMRI orientation was projected into the 2D PSOCT imaging plane and shown in the same color space as in PSOCT (Supplementary Fig. S3).

**Fig. 5.**
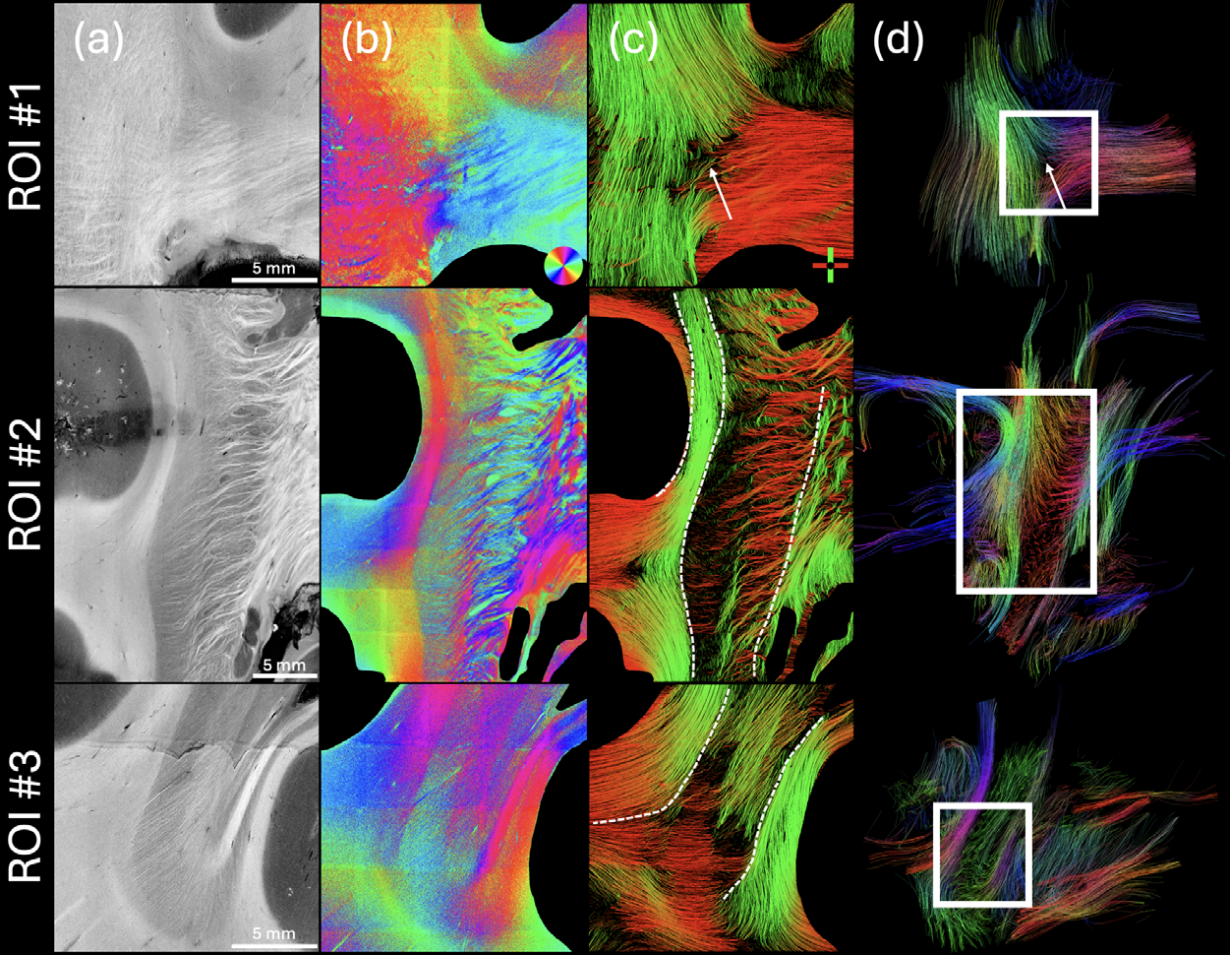
Microscopic fiber orientation and tractography. In three regions of interest (indicated inFig. 4e), images are shown for (a) PSOCT scattering coefficient map, (b) PSOCT optic axis orientation, (c) PSOCT tractography, and (d) dMRI tractography. White rectangles in (d) were enlarged in (a)-(c). White arrows: intersection areas; dotted white lines: boundaries of multiple fiber groups. The color map used to show in-plane PSOCT fiber orientation is shown in the bottom-right corner of (b). The color axis used to display the PSOCT tractography is shown in the bottom-right corner of (c), and the one used to display the dMRI tractography is shown in the upper-right corner of (d).

The high resolution of PSOCT allows us to examine detailed fiber configurations in the developing brain.Figure 5shows the fiber orientation maps of the 54-month-old in three anatomical regions along the medial side of the coronal slab, from superior to inferior, including (1) the junction of the corpus callosum and projection pathways, (2) the peri-internal capsule, and (3) the temporal stem pathways at the temporal horn of the lateral ventricle junction. Region 1 (Fig. 5, top) has three groups of large fiber bundles all coming from different directions and meeting in the center of the ROI. These three fiber bundles have clear but non-homogeneous boundaries in the orientation color map, which predicts that they intersect each other and change their layout in depth at the intersection. Similarly at the intersection (Fig. 5d, white arrow), dMRI tractography is blank (Fig. 5d, top), possibly due to a high degree of crossing. Although our current PSOCT measurements are limited to the in-plane orientation, the spatial pattern of the orientation map provides useful insights about the fiber configurations in regions where multiple fiber groups meet together. In Region 2, which covers the internal capsule and surrounding regions, multiple bands of fiber groups are elaborated (illustrated by the dotted curves), organized along the left-right axis and presented by altered orientation structures and SNR. On the left side, there are two thin layers of coherent fiber tracts with different orientations [red and green in PSOCT tractography (Fig. 5c, middle)]. In the right half, there are small tracts crossing with each other at different orientations. There is an SNR drop in the middle of the fiber tracts where the scattering coefficient map (Fig. 5a, middle) shows a dim intensity. The same region exhibits a lack of dense fiber tracts in the PSOCT tractography (Fig. 5c, middle), suggesting that fiber tracts running through the plane are missing. There is another group of fiber tracts running mostly horizontally. The rightmost band is composed of highly interwoven fibers running through the thalamus region. The dMRI tractography captures the main fiber orientation albeit with a much simpler configuration (Fig. 5d, middle). Region 3 (Fig. 5, bottom) is located where most of the fibers are oriented similarly. Despite this similarity, we still observe different fiber groups in the PSOCT tractography (Fig. 5c, bottom), as they present altered SNR as shown in the axis orientation (Fig. 5b, bottom). Low intensity on the scattering coefficient map (Fig. 5a, bottom) indicates regions where different fiber configurations are present but are not captured with the PSOCT tractography. dMRI tractography, indeed, identifies tracts running through the plane in this region (Fig. 5d, bottom).

#### 3.3 Quantitative correlation between PSOCT and dMRI

**Fig. 6.**
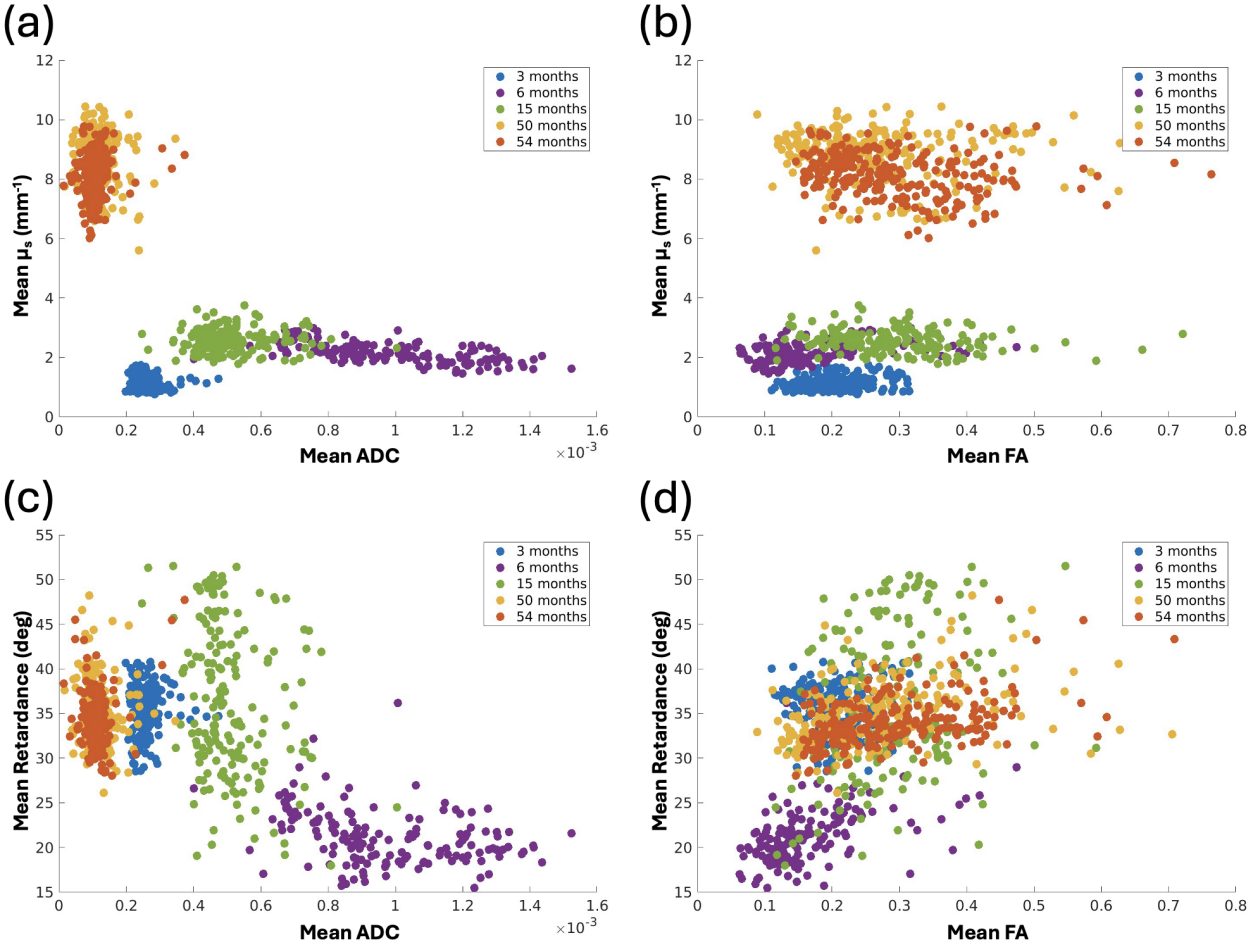
Correlation between optical properties (µ_s_and retardance) and dMRI parameters (ADC and FA) across the 5 samples of different ages. Each data point is the mean value taken from a 0.9 × 0.9 mm ROI of white matter in the aligned PSOCT and dMRI parameter maps.

As the results inSection 3.1show, the PSOCT scattering coefficient increases with age, consistent with the myelination process during development. While the same is true for PSOCT retardance increasing with myelin maturation, it is important to note that retardance can exhibit cancellation effects from crossing fibers, reducing the apparent retardance in regions with multiple fiber orientations. Previous dMRI studies have suggested that ADC values decrease, and FA values increase in early childhood (Moon et al., 2011). Similar to retardance, however, FA will also exhibit reduced values in regions with crossing fibers. In this study, we were interested in examining the correlation between PSOCT optical properties and dMRI parameter maps over the first few years of life. The scatter plots show a moderate negative correlation between scattering coefficient and ADC (r = -0.62,Fig. 6a) as well as retardance and ADC (r = -0.61,Fig. 6c), following the expected trends with myelin development. While we also observed a general increase in retardance and FA with age, the positive correlation between retardance and FA (r = 0.45,Fig. 6d) as well as scattering coefficient and FA (r = 0.29,Fig. 6b) were slightly weaker, likely due to the mixed impact in crossing fiber regions of the FA maps.

**Fig. 7.**
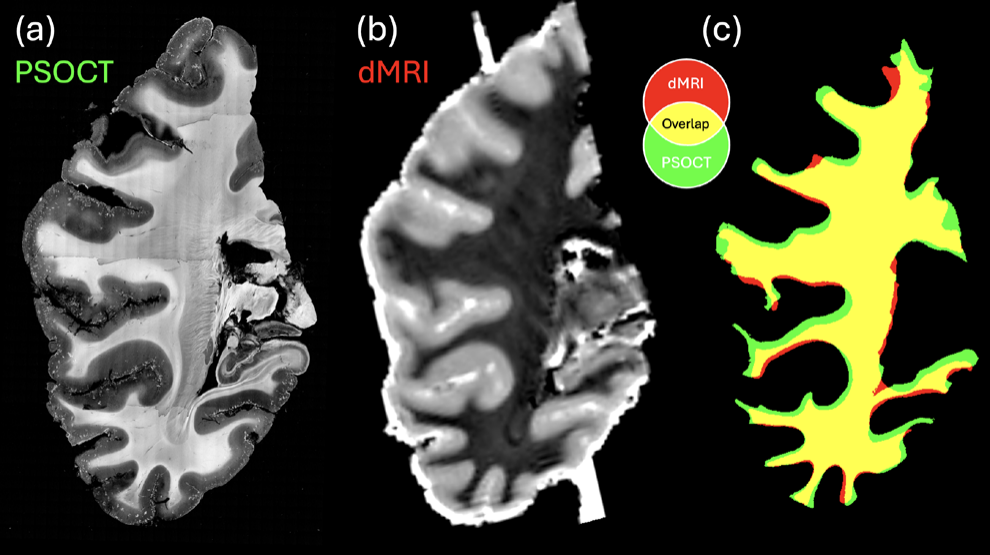
Registration between PSOCT and dMRI images for the 54-month-old sample. Registration between (a) PSOCT and (b) dMRI was performed based on their tissue and (c) white matter masks. (c) The overlap between the PSOCT and dMRI white matter masks following registration.

We also investigated the within-subject correlation of the parameter maps as a means of examining the regional variability addressed with the two imaging modalities. To make a quantitative comparison, we first assessed the quality of PSOCT and dMRI co-registration in the two volumetric images of the 50- and 54-month-old, using the Dice coefficient on both the tissue masks and the white matter masks. The Dice coefficient on tissue mask of the 54-month-old was 0.96, indicating an almost perfect overlap of the co-registered datasets. The Dice coefficient of the white matter masks was 0.87.Figure 7demonstrates the co-registration results for one section at 54 months of age. We obtained a consistent registration quality in the 50-month-old sample as well (Supplementary Fig. S4), with Dice coefficients of 0.93 and 0.75 for the tissue mask and white matter mask, respectively.

**Fig. 8.**
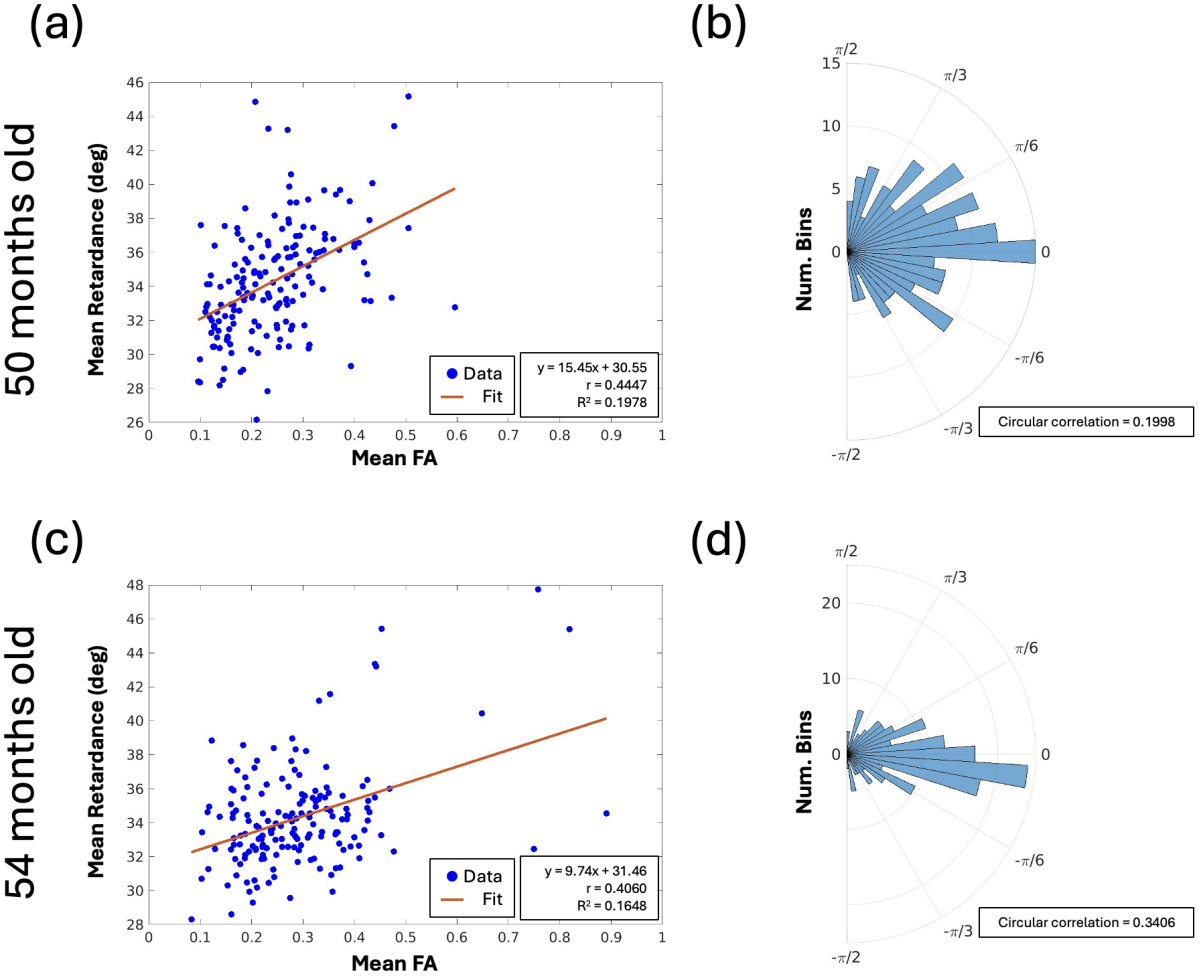
Comparison between PSOCT and dMRI parameter maps. PSOCT and dMRI images were registered for the (top) 50-month-old and (bottom) 54-month-old samples and compared in white matter. (a, c) Mean PSOCT retardance vs. mean dMRI FA. (b, d) Angular difference between PSOCT orientation and dMRI orientation.

Next, we selected 120 ROIs dispersed in the white matter of each sample and examined the relationship between PSOCT optical properties and dMRI parameter maps, including retardance vs. FA, retardance vs. ADC, scattering coefficient vs. FA, scattering coefficient vs. ADC, and PSOCT orientation vs. dMRI orientation. Among the scalar maps we examined, only retardance and FA show a moderate correlation in the white matter regions (Fig. 8a, c). At 4 years old, the two metrics generally agree on the structural anisotropy of the white matter that is higher in parallel fiber bundles and lower in crossing regions. Despite a negative correlation between scattering coefficient and ADC across developmental age (Fig. 6), such correlation within the 4-year-old brain samples is not seen. It is possible that regional differences in myelin maturation are diminished, and the two metrics have different sensitivity in capturing the local variation. The fiber orientation shows a strong agreement in major fiber bundles between the two modalities. To compare the distribution of in-plane fiber orientation angles between PSOCT and dMRI, we plotted the angular difference between the two modalities (Fig. 8b, d), which minimizes the difference in polar space for accurate representation (i.e., 178° – 1° = 3°, not 177°). The angular differences between dMRI and PSOCT are distributed in a narrow range centered at 0°, indicating a similarity of the major fiber orientation measured by PSOCT and dMRI in young children. This consistency serves as a cross-validation for the two modalities to study the connective pathways in the developing brain.

## 4 Discussion

The use of PSOCT to reveal microstructures and fiber pathways in large-scale brain tissues has received considerable attention in human neuroscience recently. The advantageous blockface imaging and label-free contrast mechanism enable histological level details in the image, while remaining free from drawbacks like staining bias and limiting tissue distortions or damage encountered in traditional histology. In this study, we leveraged the PSOCT technology to investigate the neurodevelopment of white matter during the first 5 years of life. We used a combination of PSOCT and dMRI to study five whole coronal slabs of the human brain across different ages. PSOCT clearly advocates for the use of optical properties as important biomarkers for myelination processes. The optic axis orientation delineates the direction of the fiber tracts in the infant brain at the age of 3 months, which is consistent with dMRI. The microscopic resolution of PSOCT is capable of resolving small fascicles less than 100 micrometers thick, as well as complex fiber configurations with inter-weaving and splitting. To the best of our knowledge, this is the first PSOCT study to investigate the development of the human brain from infancy to early childhood. Its combination with dMRI further enhances the analysis of fiber tracts and serves as a cross-validation in the developing brain.

### 4.1 Optical properties revealing myelin development

The myelogenic process begins around birth and continues through adolescence. Our PSOCT study shows substantial development of myelin content in five coronal slabs from 3 to 54 months of age. The optical property of scattering coefficient begins to show a higher signal in the white matter compared to the gray as early as 3 months of age, when myelination is still in its early stages and fibers are largely unmyelinated (Deoni et al., 2011;Dubois et al., 2014;Hasegawa et al., 1992). Scattering coefficient in the white matter continues to increase over the following 12 months. By 15 months of age, the contrast between white and gray matter becomes fully pronounced (Fig. 2). By 4 years of age, scattering coefficient in the white matter increases almost 10 fold (Fig. 3) to approximately 80% of the scattering coefficient reported in the adult human brain (Chang et al., 2022;Liu et al., 2022;H. Wang et al., 2017). The use of optical properties to quantify the myelin content has been validated in previous studies. A quantitative study on PSOCT and histology showed that the scattering coefficient is linearly correlated with the optical density of Gallyas stain, a traditional histological method for myelinated fibers, and this linear relationship holds across multiple brain regions and subjects (Chang et al., 2022).

It is interesting to note that regional differences were also observed in the coronal section at 6 months of age, with higher scattering coefficients in the frontal/parietal lobes than in the temporal lobe. Previous studies have reported that myelin development tends to be from posterior to anterior (Ballesteros et al., 1993;Grotheer et al., 2022) and that the occipital lobe is myelinated earlier than the frontal lobe. The present study indicates that the timeline of myelination may also differ between the border of the parietal/frontal lobes (somatosensory/motor cortices) and temporal lobes. Overall, the sensitivity of the optical properties to myelin content suggests that PSOCT may be a useful tool for studying myelination defects in neurodevelopmental disorders.

### 4.2 Connective pathways in the developing brain

Compared to traditional histology or other forms of microscopy, a distinct advantage of PSOCT is that it has inherent label-free sensitivity to measure the myelin optic axis, allowing for the quantitative determination of fiber orientations across white matter. Anisotropy in the molecular structure of myelin gives rise to birefringence along a unidirectional axis, which is parallel to the axons in the brain. Previous studies using PSOCT or polarized light imaging have shown that the optic axis orientation provides accurate quantification of fiber orientation in the cortex, deep white matter, and subcortical nuclei of the adult human brain (Larsen et al., 2007;Liu et al., 2022;H. Wang et al., 2018). In this study, we extended the application of optic axis orientation data in the developing brains with a combined dMRI technique to study the fiber pathways. The optic axis orientation is capable of delineating fiber orientations in infants as young as 3 months of age, despite the low birefringence during the mild myelination stage. Major fiber bundles showed consistency with dMRI tractography for in-plane orientation, during the first 5 years of life. We noticed that noise in the optic axis orientation map is elevated in samples of young age (3 and 6 months old), possibly due to the low birefringence of the white matter. Since the noise in orientation measurements is inversely related to the SNR of the intensity signal (Everett et al., 1998), low birefringence would be expected to lower the SNR of the cross-polarization channel that affects the overall orientation measurements. Nevertheless, the optic axis orientation is a useful tool for studying fiber pathways in the developing brain.

The microscopic resolution of PSOCT reveals complex fiber configurations at finer scales, such as interwoven fibers in the internal capsule and the multiple fiber bands adjacent to it (Fig. 5). The different orientations of these small tracts are appreciated by color-coding the optic axis, which would be lost in the homogenous intensity of scattering otherwise. Tractography applied to the optic axis orientation map differentiates the clusters of fiber bundles running in different directions. One limitation of the current PSOCT technology is that the optic axis depicts only 2D orientation information, which is the projection of the 3D axis into a plane perpendicular to the illumination light. Unlike dMRI tractography that captures the 3D tract orientation, the through-plane angle of fibers is not captured with PSOCT. To map complete connectivity, further advancement of PSOCT technology requires 3D axis orientation measurements, which can be achieved with multiple illumination angles (Liu et al., 2016;Ugryumova et al., 2006,^2009^;Y. Wang et al., 2016).

### 4.3 Correlation between PSOCT and dMRI images in brain development

dMRI provides valuable information in identifying coherent fiber pathways using diffusion properties in the fiber tracts. Using tractography techniques, pathways throughout the white matter of the brain can be depicted in three dimensions (Tuch et al., 2002;Vasung et al., 2019). Our study takes advantage of multi-modality techniques to examine the developing brain across scales. Importantly, we use both PSOCT with 10 µm resolution and ex vivo dMRI with 800 µm resolution, spatially co-registered, to investigate the entire coronal section. We found that both optical properties and dMRI properties reveal developmental patterns in the white matter that are generally correlated over the first 5 years of life (Fig. 6). The optical properties of scattering coefficient and retardance are, in general, negatively correlated with ADC and positively correlated with FA. One exception is the low ADC value for the very young brain sample of 3 months old. Normally, ADC values decrease with age in in vivo MRI of healthy brains (Löbel et al., 2009). However, our ex vivo fixed brain MRI data in this study showed that the brain younger than 6 months of age had lower ADC values than older brains. This trend is consistent with our other data not presented in this paper. A possible reason for this would be a combination of multiple factors, including that brain tissue is immature at less than 6 months of age with no or lesser degrees of myelination in the white matter, axons/dendrites are overrepresented before pruning, and their pathways are not well organized (Deoni et al., 2011;Huang et al., 2013;Natu et al., 2021). In general, formalin fixation tends to reduce the amount of water in the tissue, and the structure of unmyelinated excess brain fibers at younger than 6 months of age may tend to become tight and denser when water content is reduced (Sun et al., 2003;Weisbecker, 2012;Yoshimaru et al., 2024). PSOCT and dMRI fiber orientation presents a consistent measurement in major fiber bundles despite the significantly differing resolutions. As revealed in the 4-year-old samples, their angular difference is centered around 0° in co-registered images (Fig. 8). A closer within-subject examination of the same samples exhibits a moderate correlation between PSOCT retardance and dMRI FA, indicating a general agreement in measuring the coherency of fiber configurations. Similar findings in the retardance and the orientation have been reported in previous studies on adult human brains (Jones et al., 2020;H. Wang, Zhu, Reuter, et al., 2014). The regional variability in the developing brain warrants further investigation in both optical and diffusion measurements. Our new technology opens up great possibilities for the study of normal neurodevelopment of the brain and neurodevelopmental disorders.

### 4.4 Challenges and future directions

There are several challenges in postmortem human brain imaging. Firstly, tissue quality is not easy to control due to the scarcity of brain samples, which is particularly a problem in infant brains. The cause of death may not be identified, leaving the brain condition unknown. Additionally, long postmortem intervals, improper storage conditions, and other tissue processing steps may further degrade myelin integrity, which becomes a confounding factor when assessing myelin development with dMRI and PSOCT. Other challenges that come with this type of work, involving high-resolution, high-throughput, volumetric imaging, is the huge amount of data that is generated. The raw PSOCT data could reach hundreds of TB for one scan of the coronal slab. In the future, advanced computational resources and data storage solutions will be imperative to studying brain development in large cohorts.

## Supplementary Material

Supplementary Material

## Data Availability

All software and procedures concerning the data acquisition and analysis have been detailed in the Materials and Methods section. All data sets are available from the corresponding authors upon reasonable request. We will evaluate the request case by case, consulting with the Brain Bank.
