# Supplementary material for: Imaging of developing human brains with ex vivo PSOCT and dMRI
Source: Imaging Neurosci (Camb). 2025 Mar 24;3:imag_a_00510. doi: 10.1162/imag_a_00510 (PMC12319849; doi:10.1162/imag_a_00510)
Supplement: Supplementary Material [file imag_a_00510-supp.pdf]

Supplementary Material

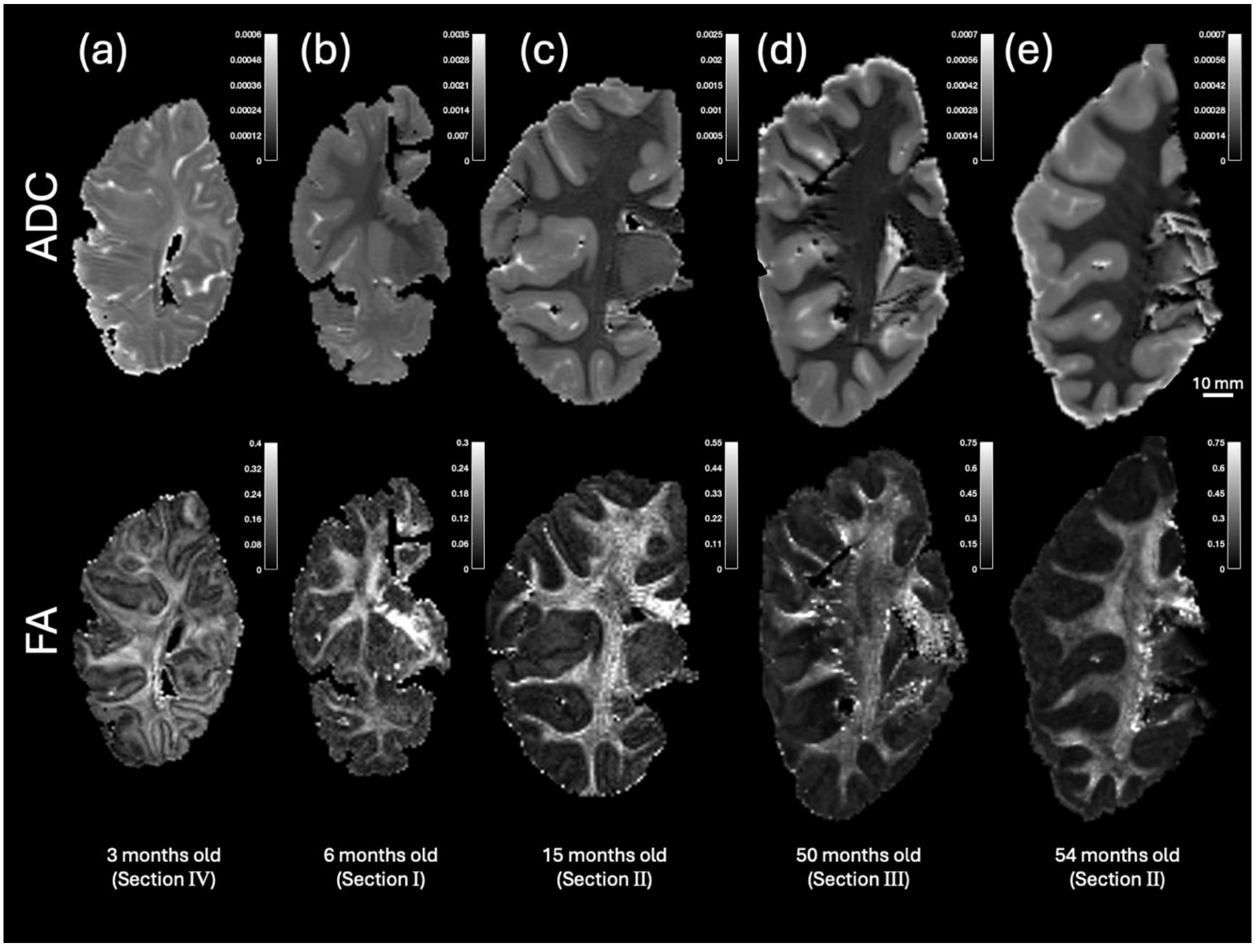

Fig S1. dMRI parameter maps of apparent diffusion coefficient and fractional anisotropy showing development in the first 5 years. (top) Apparent diffusion coefficient (ADC) maps and (bottom) fractional anisotropy (FA) are shown for five samples from infancy to early childhood: (a) 3 months old, (b) 6 months old, (c) 15 months old, (d) 50 months old, (e) 54 months old. The scale for each grayscale image is shown in the top-right inset of each panel. The anatomical location of each slab is denoted by Roman numerals and is shown in Fig. 1. These dMRI parameter maps correspond with the PSOCT optical property maps shown in Fig. 2.

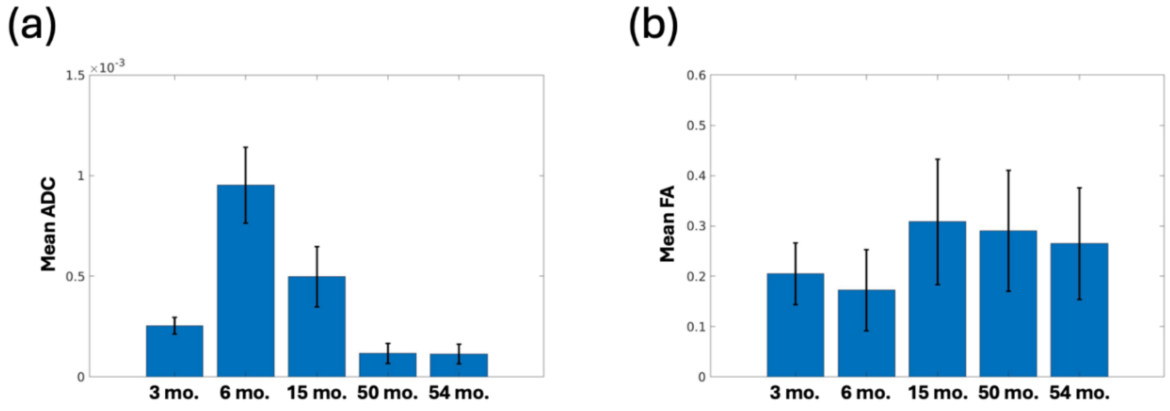

Fig S2. Change of dMRI parameters with white matter development. (a) Mean apparent diffusion coefficient (ADC) and (b) mean fractional anisotropy (FA) analyzed across all pixels in white matter. The error bars show standard deviation. These dMRI bar plots correspond with the PSOCT bar plots shown in Fig. 3.

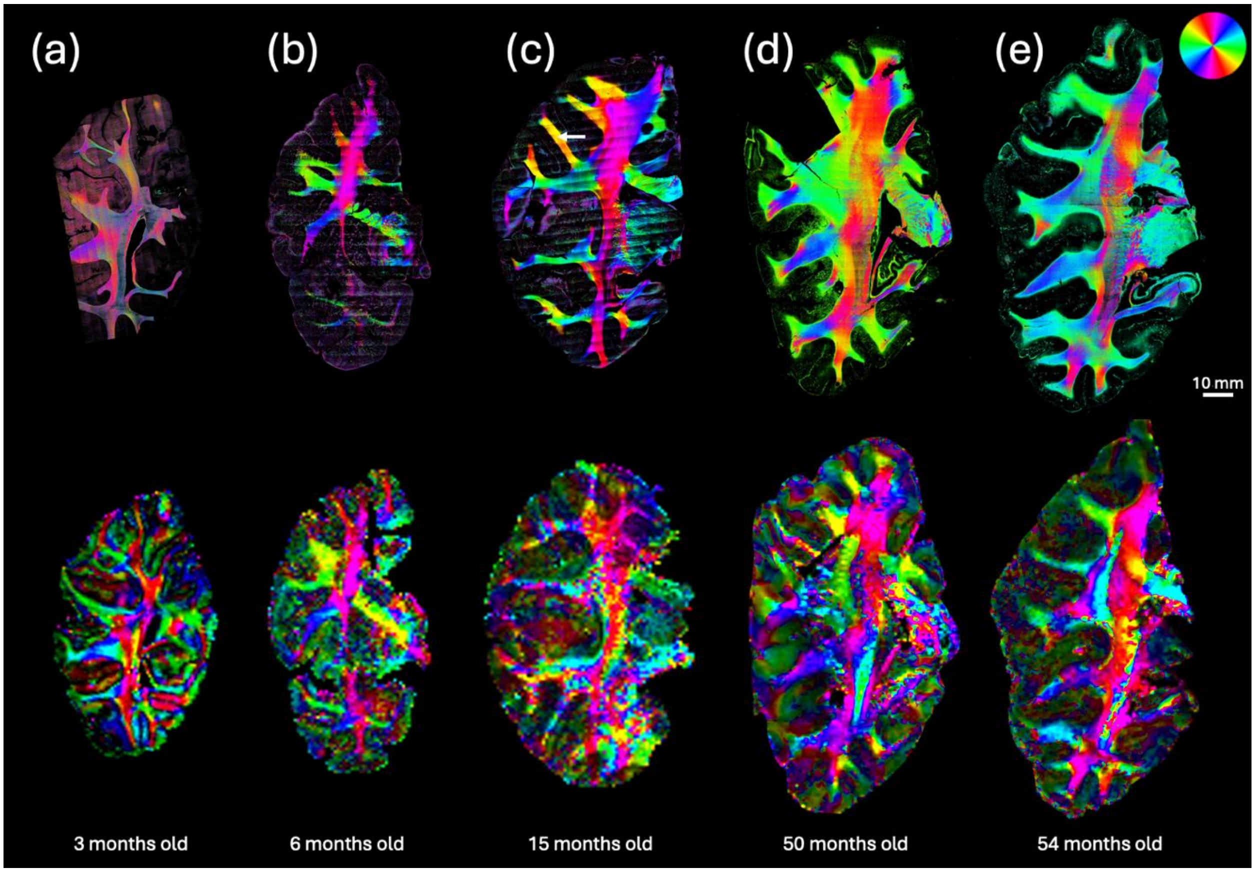

Fig S3. PSOCT optic axis orientation maps and dMRI tractography visualized as 2D orientation maps. (top) PSOCT optic axis orientation maps and (bottom) dMRI 2D orientation maps are shown for five samples from infancy to early childhood: (a) 3 months old, (b) 6 months old, (c) 15 months old, (d) 50 months old, (e) 54 months old. The color scale used for showing the fiber orientation for both modalities is shown in the top-right corner of (e). This representation of the dMRI orientation is an alternative to the visualization of the orientation data shown in Fig. 4.

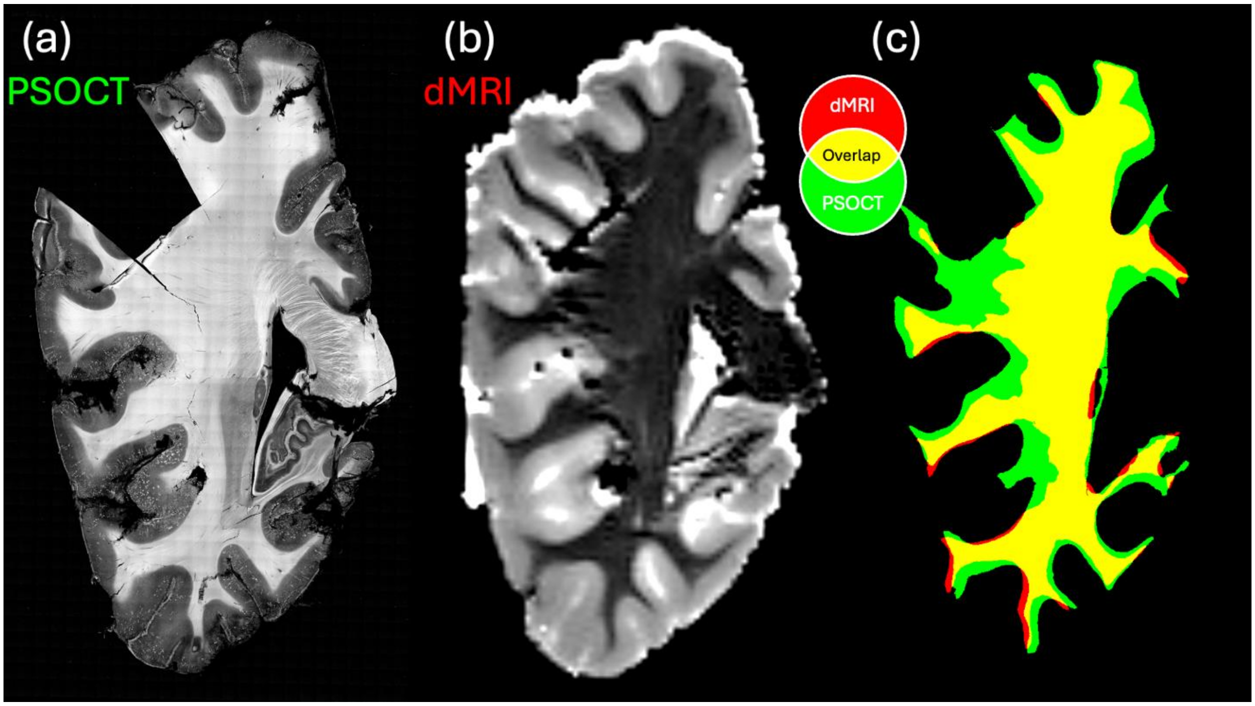

Fig S4. Registration between PSOCT and dMRI images for the 50-month-old sample. Registration between (a) PSOCT and (b) dMRI was performed based on their tissue and (c) white matter masks. (c) The overlap between the PSOCT and dMRI white matter masks following registration. This registration diagram pairs with the similar version shown for the 54-month-old sample in Fig. 7.

Supplementary Table 1: Detailed individual information about the specimens

	Brain age (years)	Disease/ Cause of death	Sex	Race	Postmortem interval (hours)	Additional information about the cases
**Case 1, #5308**	4 years and 183 days old	Blunt force head injuries	Male	Caucasian	21	He was a children with Autism Spectrum Disorder (ASD). The cause of death was skull fractures as a result of blunt force head injuries resulting as a pedestrian hit by car. His neuropathology report described blunt force head injuries, multifocal subarachnoid hemorrage, intermediate contusions, cerebral white matter tracts and and brainstem, possible microdysgenesis, temporal lobe (clinical diagnosis of Autism).
**Case 2, #6032**	4 years and 51 days old	Head and neck injuries	Male	Caucasian	25	Cause of dead was head and neck injuries due to traffic accident. His neuropathology report included subarachnoid hemorrage in right lateral frontal lobe and ambient cistern.
**Case 3, #4403**	202 days old	Undetermined	Female	Caucasian	4	Cause of death was undetermined. The final neuropathology report included unremarkable infant brain.
**Case 4, #4455**	1 years and 100 days old	Drowning	Female	Caucasian	39	Cause of death was drowning in swimming pool. Her neuropathology report included unremarkable infant brain. Her development was appropriate for the staged age.
**Case 5, #5995**	103 days old male	Sudden unexpected death of infancy	Male	NA	22	(race was not applicable) Cause of death was sudden unexpected death of infancy. His report included development was was appropriate for staged age.
**Case 1, #5308**	4 years and 183 days old	Blunt force head injuries	Male	Caucasian	21	He was a children with Autism Spectrum Disorder (ASD). The cause of death was skull fractures as a result of blunt force head injuries resulting as a pedestrian hit by car. His neuropathology report described blunt force head injuries, multifocal subarachnoid hemorrage, intermediate contusions, cerebral white matter tracts and and brainstem, possible microdysgenesis, temporal lobe (clinical diagnosis of Autism).
